# Supplementary material for: A comparative national-level analysis of government food system resilience activities across four developed countries at varying stages of planning
Source: BMC Public Health. 2024 Feb 14;24:461. doi: 10.1186/s12889-024-17919-x (PMC10865510; doi:10.1186/s12889-024-17919-x)
Supplement: Supplementary file 2 — Additional file 2. R Code for Analysis. [file 12889_2024_17919_MOESM2_ESM.pdf]

Supplementary Information: File 2

R Code for Analysis

```
################################################################################
# Comparing food resilience response actions by country according to response
# coding in Lloyd et al. (2023).
#
# This code is intended to analyze individual country actions across the
# resilience categories
#
# Code prepared by Lyndsey Dowell for research conducted in Lloyd et al. (2023)
# Last Updated: January 2023
################################################################################

#install.pakages("dplyr")
library(dplyr)

# Set data directory

# Read in issue and action data
raw <- read.csv("FSR_Coding.csv")

  # Replace NAs with 0 - recommended step
  # raw <- raw %>%
```

```
  # mutate_at(c(4:47), ~replace(., is.na(.), 0))

# Read in categorization for issue and action coding
cat <- read.csv("cat_ids.csv")

# Subset action data for analysis

cat_list <- unique(cat[,1])

data_list <- list()

 # Loop through to create data frames for each analysis category
 for(i in 1:length(cat_list)){
   df <- raw[,c(1,4)]
   c = 2
   for(j in 1:nrow(cat)){

     if(cat[j,1] == cat_list[i]){
       c = c + 1
       df <- cbind(df,raw[,which(colnames(raw) == cat[j,2])])
       colnames(df)[c] <- as.character(cat[j,2])
     }
   }
   data_list[[length(data_list)+1]] <- df
   names(data_list)[i] <- as.character(cat_list[i])
 }
```

```
# Function to create actions pivot table
pivot <- function(data){
   table <- data %>%
    filter(ACTION == 1) %>% # Only looking at actions
    group_by(COUNTRY) %>% # Grouping results by country
    summarize_at(c(1:(ncol(data)-1)),sum, na.rm = TRUE) # Calculating action frequency
}

# Create and export pivot tables for each analysis category
for(i in 1: length(data_list)){
  pv <- pivot(data_list[[i]])
  write.csv(pv, paste(as.character(cat_list[[i]]), 'pivot', 'csv', sep = '.'))
}


##############################################################################
# Looking for repeated action attribute combinations across and within countries
# (Lloyd et al., 2023).
#
# This code is intended to count the repetitions of identical attribute
# combinations across all resilience categories
#
# Code prepared by Lyndsey Dowell for research conducted in Lloyd et al.(2023)
# Last Updated: January 2023
##############################################################################
```

```r
#install.packages("dplyr")
#install.packages("tidyr")
library(dplyr)
library(tidyr)

# Set data directory

# Read in issue and action data
df <- read.csv("FSR_Coding.csv")

# Narrow list down to just actions
df2 <- df %>% drop_na(ACTION)

# Look for combinations of a subset of attributes

  # Combination set 1: look at frequency of identical action coding across all
  # countries to identify where countries have the same responses
  ## (remove country, policy descriptors, time frame, and other descriptors)

  combo_all <- df2[,c(5:13,15:25,26:32,34:39)] %>% #sub-setting to desired attributes
    group_by_all() %>%
    summarise(count = n()) %>%
    arrange(desc(count))

    # Assign combination ID
```

```
  combo_sub1 <- combo_sub1 %>%
    mutate(ID = row_number())

  # Combination set 2: include country in subset to see frequencies of identical
  # action coding specific to individual countries
  ## (remove policy descriptors, time frame, and other descriptors)

  combo_by_country <- df2[,c(1,5:13,15:25,26:32,34:39)] %>% #sub-setting to desired attributes
    group_by_all() %>%
    summarise(count = n()) %>%
    arrange(desc(count))

## Export results tables
write.csv(combo_all, "attribute_combinations_altogether.csv")
write.csv(combo_by_country, "attribute_combinations_individual.csv")
```
